# Autogenous Bone and Bioactive Glass around Implants Placed Simultaneously with Ridge Splitting for the Treatment of Horizontal Bony Defects: A Randomised Clinical Trial

**DOI:** 10.1155/2021/2457328

**Published:** 2021-07-28

**Authors:** Walid Elamrousy, Mohamed Osama, Dalia Rasheed Issa

**Affiliations:** ^1^Periodontology Department, Faculty of Dentistry, Kafrelsheikh University, Kafr El-Sheikh 21614, Egypt; ^2^Clinical Implantology Department, Faculty of Dentistry, Alexandria University, Alexandria 17862, Egypt

## Abstract

**Objective:**

To compare using autogenous bone with or without bioactive glass in ridge splitting of horizontal bone defects combined with simultaneous implant placement.

**Materials and Methods:**

In control group, bone expansion was performed and autogenous bone was used to augment the intercortical bone defect. In study group, autogenous bone was mixed with bioactive glass (1 : 1 in volume). In both groups, the implants were inserted simultaneously with ridge splitting. Six months following implant insertion, bone width and height were evaluated. Statistical analysis utilizing paired Student's *t*-test was used for comparing results within the same group, whereas independent samples *t*-test was used for intergroup variables comparison.

**Results:**

The mean bone width and labial and mesiodistal crestal bone height values were increased significantly in both groups from baseline to 6 months postoperatively. Comparing the two groups showed nonstatistical significant difference regarding the labial crestal bone loss, while the ridge width gain values were significantly higher in the study group than in the control group. The mesiodistal bone loss was significantly higher in control group than in study group.

**Conclusion:**

Autogenous bone was mixed with bioactive glass (1 : 1 in volume) to fill intercortical defect created after ridge splitting to decrease peri-implant bone resorption associated with autogenous bone alone. This trial is registered with clinical trial registration: NCT04814160.

## 1. Introduction

The use of dental implants for prosthetic rehabilitation may be complicated by hard- and soft-tissue deficiencies caused by trauma, tumor, infection, loss of teeth, or periodontal destruction. Those deficiencies may prevent proper implant placement, adversely affecting osteointegration and esthetic outcomes especially in anterior teeth [1].

Various treatment modalities could be used for alveolar ridge reconstruction such as distraction osteogenesis, block bone grafts, and guided bone regeneration [2]. The disadvantages of the previously mentioned techniques include soft-tissue dehiscence, collapse or instability of membrane, bone loss, donor site morbidity, increased treatment cost and healing time, and inadequate quality of the newly formed bone [2].

Alveolar ridge split approach was first presented in 1984 by Tatum for maxillary bone augmentation [3] and was modified by Simion et al. [4] and by Scipioni et al. [5].

The defect created during the ridge splitting procedure was described as a “self-space making” defect and was considered as a four-wall intrabony box. This box can be filled with different grafting materials; therefore, ridge splitting is more effective for lateral augmentation of narrow ridge than block onlay grafting, membrane-protected blocks, guided bone regeneration, and interpositional grafts. Furthermore, ridge splitting technique had an added advantage by allowing implant placement simultaneously with augmentation [4].

The applied bone graft affects the success of ridge splitting. Although autobone grafts are still considered to be the gold standard due to the osteogenic and osteoinductive potential [6], only a limited amount of autogenous bone can be obtained from intraoral donor site. Alloplastic bone grafts, especially bioactive glass, could be an alternative material to be mixed with autogenous grafts for the treatment of bone defects. Bioactive glass bone substitute is biocompatible and easy to be handled and has haemostatic and osteoconductive characteristics and potential osteoinductive function. Bioactive glass stimulates osteogenesis by promoting osteoblasts to utilize the adsorbed proteins and form mineralized extracellular matrix, thus allowing rapid new bone formation [7].

No controlled trials have been performed to prove the benefits of bioactive glass as a bone graft substitute with ridge splitting technique. To the best of our knowledge, this is the first study that will evaluate the effect of autogenous bone mixed with bioactive glass in ridge splitting with simultaneous implant placement. The present study was conducted to evaluate the clinical and radiographic outcomes following the application of the alveolar ridge splitting and simultaneous implant placement approaches using autogenous bone with or without bioactive glass, in patients with horizontally atrophic jaw bones in maxillary esthetic zone.

## 2. Materials and Methods

### 2.1. Study Design and Hypothesis

The participants and data collection examiner were blinded to the study. The present study was randomized parallel two‐group design clinical trial (NCT04814160). The Faculty of Dentistry, Kafrelsheikh University research ethical committee approved the study (KD/06/20). The study was conducted at the Periodontal Department, Faculty of Dentistry, Kafrelsheikh University, Egypt, and surgical interventions and data collection occurred between April 2020 and March 2021. All recruited participants did not afford any treatment costs. The current study procedures were explained to all participants, and they agreed to participate in this study and signed a consent form.

This present study tested two hypotheses: (1) the first is the hypothesis of the superiority of a (1 : 1 in volume) mixture of autogenous and bioactive glass bone graft over autogenous bone graft alone (i.e., better than) and (2) further, if the first hypothesis was rejected (i.e., a (1 : 1 in volume) mixture of autogenous and bioactive glass bone graft is not superior to autogenous bone graft alone), we tested the noninferiority hypothesis of the autogenous bone graft alone (i.e., not worse than) according to an acceptable limit of noninferiority.

### 2.2. Study Sample

**Figure 1:**
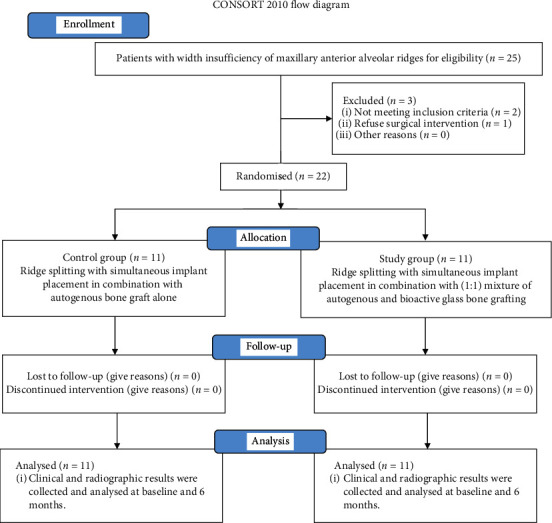
Flow diagram for the participants in the current study.

The target population had inadequate bone volume for implant placement due to width insufficiency of maxillary anterior alveolar ridges, aged from 18 to 50 years, and patients were retrieved from the Outpatient Clinic of the Department of Oral Medicine and Periodontology, Faculty of Dentistry, Kafrelsheikh University. No gender restrictions were considered for initial screening (Figure 1).

Eligible participants should present good general health and agree to random assignment to any of the two parallel study groups. Participants had minimum 3 months as postextraction healing period and horizontal maxillary anterior ridge defects with at least bone width of 3 mm and bone height of 13 mm. The criteria of patients' exclusion were vertical ridge defect; undercut on the labial/buccal side; thick cortical bone without cancellous bone inside; uncontrolled systematic disorders or diabetes mellitus; uncontrolled periodontal disease; history of head and neck radiotherapy; smokers; pregnancy; noncompliant patients; allergy to the used medications; uncooperative individuals or those unable to attend the study follow-up appointments.

Sample size calculation was undertaken via G power version 3.1 statistical software based on the following preestablished parameters: an alpha-type error of 0.05, a power test of 0.80, and a total sample of 22 subjects (11 subjects for each group), which appeared to be sufficient.

### 2.3. Presurgical Therapy and Group Randomization

**Figure 2:**
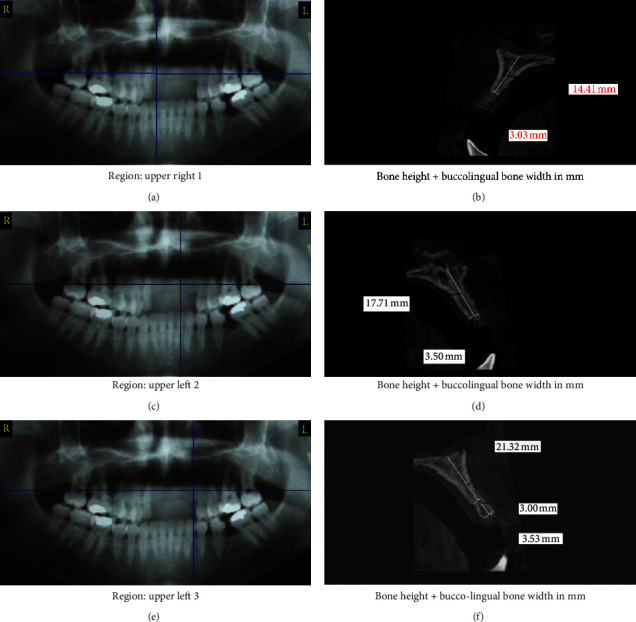
Preoperative CBCT.

Presurgical therapy consisted of a thorough full-mouth scaling and root planning. Patients were randomly assigned immediately before surgery into two groups (11 patients each) by computer‐assessed randomization software (Random Allocation Software, version 1.0). Control group received ridge splitting with simultaneous implant placement in combination with autogenous bone graft alone, while study group used 1 : 1 mixture of autogenous and bioactive glass bone grafting. Bone volume was assessed two weeks before operation using CBCT (Scanora 3D, Soredex Oy, Tuusula, Finland) (Figure 2). Each participant's assignment group was labelled and then concealed in black sealed envelopes to avoid selection bias. The two groups were identified only after data collection.

### 2.4. Surgical Procedure

**Figure 3:**
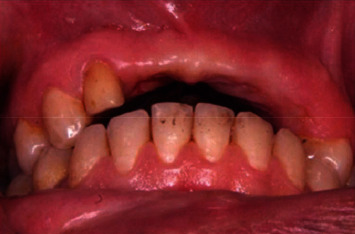
Preoperative view.

**Figure 4:**
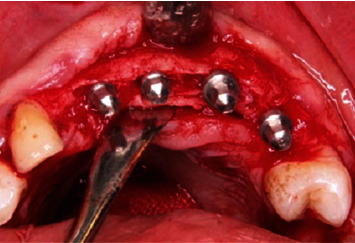
Expansion of osteomised ridge using bone wedges.

Surgery was done under local anesthesia (Figure 3). Midcrestal incision was followed by reflection of full thickness flap. Midcrestal cut without vertical osteotomy was done using piezosurgery unit (Piezotome® Cube, Acteon Group Ltd., England), and then the cut was extended deep to the implant length. The ridge was expanded progressively using bone wedges (Microdent Implant System, Spain) (Figure 4). Dental implant fixture (3.75^*∗*^13 mm Humana Dental Implants & Accessories GmbH, Germany) was placed stably with 1 mm minimal thickness of buccal bone plate.

### 2.5. Autogenous Bone Harvesting

**Figure 5:**
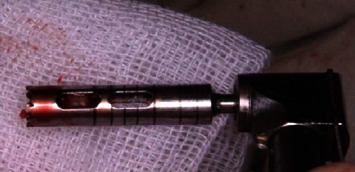
The harvested autogenous bone using trephine bur.

Incision was made at mucogingival junction in the symphysis area followed by full thickness flap reflection, and then corticocancellous bone was harvested using a trephine drill (Figure 5). Then the harvested bone was crushed using manual bone crusher with titanium teeth (Jullundur Surgical Works, Mumbai, India). Finally, the flap was sutured by interrupted and horizontal mattress suture by 3-0 black silk.

**Figure 6:**
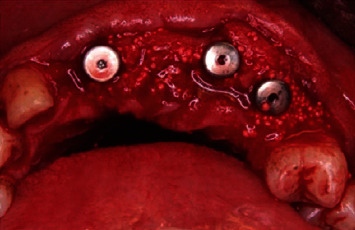
The 1 : 1 mix of bioactive glass and autogenous bone around dental implants.

In control group, harvested autobone graft was applied in the gap between buccal and lingual plates. Study group received the same way of treatment except using (1 : 1 in volume) mixture of the harvested autogenous bone with bioactive glass bone graft (BonyGlass, Pharma-Excellence, Egypt) (Figure 6). Surgical site was completely closed and wound edges were sutured in a tension-free way. The same operator performed all of those surgical procedures for all participants.

### 2.6. Postoperative Care

Patients were prescribed amoxicillin‐clavulanic acid 1 g every 12 hours, antibiotic for 7 days (Augmentin 1 g, SmithKline Beecham Pharmaceuticals), and 0.12% chlorhexidine hydrochloride mouth wash twice daily (Hexitol, ADCO, Cairo, ARE) for 10 days. Sutures were removed 14 days postsurgically. No removable denture was allowed for 2 weeks.

Six months postoperatively, clinical evaluation was recorded including implant survival and presence of infection, pain, tenderness, or wound dehiscence. CBCT was taken at 6 months postoperatively to evaluate ridge width and bone level. All clinical and radiographic measurements were measured and recorded by one calibrated masked examiner.

Participants were assessed at baseline before surgery and 6 months postoperatively. Primary outcomes included the presence of pain, implant mobility, and implant survival rates. The secondary outcome variable measures bone changes including bone height and width using OnDemand3D™ App-3D CBCT software (Cybermed, CA, USA). All radiographic recordings were measured using fixed anatomical landmarks at baseline and different follow-up periods. The alveolar ridge width was measured buccolingually in axial view 2 mm apical to the implant collar margin. Mesiodistal vertical bone height was measured using coronal view from a fixed anatomical landmark point to the mesial and distal marginal bone level. Moreover, sagittal view was used to measure buccolingual vertical bone height extending from fixed anatomical reference point to the buccal and lingual alveolar crest.

### 2.7. Data Analysis

Data analysis used IBM SPSS V20 software (IBM Corp., Armonk, NY, USA). Paired Student's *t*-test was used to compare the obtained results within the same group of patients at 2 different intervals. The independent sample *t*-test was utilized to compare variables between the two groups. If *p*-value was <0.001, the test was considered significantly different.

## 3. Results

### 3.1. Study Data

Horizontal alveolar ridge augmentation by using the ridge splitting technique was performed between April 2020 and March 2021. 30 implants were inserted in 22 patients (14 males and 8 females), with an average age of 37 years (range 21–50 years) (Table 1).

All patients fulfilled the inclusion and exclusion criteria of the present study. All patients were randomly divided into two groups, each including 11 patients. The two groups underwent ridge splitting technique accompanied by simultaneous implant placement utilizing either autogenous bone graft (control group) or (1 : 1 in volume) mixture of autogenous and bioactive glass bone grafting material (study group).

### 3.2. Clinical Results

Wound healing of the augmented region was uneventful in all patients, and no signs of local persistent pain, tenderness, or wound dehiscence were observed throughout the evaluation period. Four patients presented with mild pain and mild local swelling within the first 2 days after surgery, which gradually receded within 6 days postoperatively.

Implants showed no mobility all over the evaluation period. The survival rates of all implants were 100%.

### 3.3. Radiographic Results

Postoperative CBCT at 6 months after surgery revealed a substantial increase in the alveolar ridge width after splitting. Moreover, the average preoperative alveolar ridge width was 4.05 ± 0.13 mm and 3.72 ± 0.15 mm in control group and study group, respectively, and significantly increased to 7.67 ± 0.14 mm and 10.14 ± 0.35 mm for control and study groups, respectively, as *p* < 0.001. After 6 months postoperatively, the mean bone gain in the control group was 3.61 ± 0.42 mm, whereas the mean bone gain in the study group was 6.42 ± 1.38 mm. Upon comparing the two groups, the study group demonstrated statistically significant increase of alveolar ridge gain values when compared to control group at 6 months postoperatively as *p* < 0.001 (Table 2).

The results of our study revealed that the mean mesiodistal bone level in the control group was found to be reduced from 17.12 ± 2.30 mm preoperatively to 15.74 ± 2.42 mm after 6 months, while in the study group it was reduced from 17.70 ± 0.76 mm preoperatively to 16.67 ± 0.76 mm 6 months postoperatively. The mean mesiodistal bone levels in both groups showed statistical significant reduction at 6 months when compared to preoperative levels as *p* < 0.001. The mean mesiodistal vertical bone loss 6 months after ridge splitting was 1.37 ± 0.09 mm and 1.02 ± 0.02 mm for the control and study groups, respectively. Upon comparing both groups, the mesiodistal bone resorption of study group was significantly less than that of the control group as *p* < 0.001 (Table 3).

In the present study, the mean labial crestal bone height in the control group preoperatively was found to be 17.12 ± 0.59 mm, while in the study group it was 17.70 ± 0.76 mm. The mean labial crestal bone height in the control group 6 months postoperatively was reduced to 15.56 ± 0.63 mm and in the study group was reduced to 16.18 ± 0.8 mm. According to these results, a statistical significant vertical loss of the labial alveolar bone height was observed in both groups at 6 months postoperatively when compared to preoperative height as *p* < 0.001. Upon comparing the labial alveolar bone resorption of the 2 groups at 6 months postoperatively, there was no statistical significant difference between the two groups as the mean labial bone resorption of the control group was found to be 1.55 ± 0.10 mm, while in the study group it was 1.51 ± 0.07 mm as *p* < 0.001 (Table 4).

## 4. Discussion

Onlay and inlay bone grafts, sandwich osteotomies, guided bone regeneration, and alveolar distraction osteogenesis are the traditional methods for horizontal bone augmentation. These methods necessitate extensive periods for bone consolidation before implant insertion, which may result in donor site morbidity, unpredictable bone graft resorption rate, risk of membrane exposure, and postoperative infection [8].

Ridge splitting is a technique that creates an implant bed through performing a longitudinal osteotomy in the alveolar ridge followed by lateral repositioning of the buccal bone using a greenstick fracture. Due to thin cortical plates and fine medullary bone, ridge splitting technique is more suitable and convenient for the maxilla than the mandible. Ridge splitting and expansion procedure is efficient for the reconstruction of horizontal alveolar bony defects [9]. Moro and colleagues (2017) showed that treatment of horizontal alveolar bone deficiencies with alveolar split osteotomy was more predictable and reliable than onlay bone grafting and guided bone regeneration [10].

Conventionally, ridge splitting was performed using chisels and mallets, and then rotating and oscillating instruments were introduced in the 2000s [11]. Recently, piezosurgery has been introduced for use in split osteotomy and simultaneous bone expansion [12]. The bony cutting procedures in the current research were done using piezosurgery. The main advantages of piezosurgery when compared to other splitting techniques are the lower risk of damaging anatomically vital structures, reduction of heat generation, minimal postoperative bone loss, reduced operation time, no hazards of soft tissue damage, and psychological acceptance by the patients [12].

Ridge splitting with simultaneous implant insertion has been successfully applied over 20 years ago due to achieving immediate gain of bone width for immediate implant placement in cases with at least 3 mm of buccolingual ridge diameter. This guarantees that there is at least 1 mm of cancellous bone between buccal and lingual cortical plates, as well as 1.5 mm of compact and cancellous bone on both sides of the split ridge, allowing bony expansion and maintenance of blood supply [13, 14].

Within the present study limitations, dental implants were simultaneously placed with ridge split procedure to shorten the edentulism interval, reduce the duration of treatment, and decrease the overall costs of implant treatment without the need of second surgery compared with staged approaches [14]. Anitua and colleagues [15] and Crespi and coworkers [16] confirmed the findings of the present study that simultaneous approach had minimal intraosseous complication with high success rate.

The ridge splitting technique in the present study was performed without cutting bone vertically in maxillary esthetic zone. This goes with findings by Ehab and Hanan [17] and Kumar and colleagues [18] who documented that splitting is easily performed in the maxillary ridge without performing vertical bone osteotomy due to the quality and physical features of bone. Moreover, high predictability of the clinical outcomes of maxillary ridge splitting is due to the elasticity and flexibility of the spongy cancellous bones that allows atraumatic bone expansion.

The intrabony defect created by expansion of the osteomised ridge within the two bony plates could be supported with bony substitutes to enhance bone regeneration and implant osseointegration. Furthermore, such grafts act as a scaffold for preventing the expanded cortical plates from collapsing of and accelerating bony regeneration. In the current trial, autogenous bone was used alone in the control group whereas in the study group it was mixed with bioactive glass to fill the osteomised intercortical defect. Though autogenous bone substitutes are widely considered the “gold standard” for osseous reconstruction due to their osteogenic, osteoinductive, and osteoconductive characteristics, some drawbacks upon clinical application were detected as morbidity of the donor region, the need for second surgery, surgery under general anesthesia, etc. [19].

Mixing of bone substitutes to the autogenous bone, as performed in the study group of our study, has been proposed by some authors to boost the graft volume with harvesting minimal autogenous bone from the donor areas. In addition to these positive outcomes, the osteoinductive characteristics of autogenous bone were added to bone substitutes and improve the predictability of long-term resorption [20].

In our study, an alloplastic bone substitute, bioactive glass, was mixed with autogenous bone for the augmentation of the split ridge. Bioactive glass has many advantages such as its cohesiveness and graft retention characteristics, lack of immune responses and infection transmission, simple manipulation during surgery, and minimal susceptibility to infection because of its hydrophilic property that allows antibiotic penetration within the graft [21]. Bioactive glass has an osteoconductive property through binding by chemical adhesion with the newly formed bone [22]. This occurs as a result of tissue fluids corroding the glass surface, stimulating a layer of calcium rich in phosphorus and a sublayer of silica to attach closely to the bony apatite crystals. Studies have shown encouraging outcomes with bioactive glass in periodontal problems and maxillary sinus bone augmentation due to these features [22].

No clinical studies have been done to assess using bioactive glass alone or a mixture of bioactive glass and autogenous bone with ridge splitting approach. Few researches have been undertaken to evaluate the newly formed bone in maxillary sinus augmentation and intrabony periodontal lesions utilizing bioactive glass mixed with autogenous bone [7, 21, 23]. Menezes et al. [21] mixed bioactive glass with autobone graft for maxillary sinus augmentation, and the results revealed more bone deposition and superior preservation of the graft volume after 6 months when compared to using only autobone graft. In 2017, Pereira et al. [23] compared the newly formed bone and cells' behavior upon using autogenous bone alone, a 1 : 1 bioactive glass to autogenous bone substitutes, and bioactive glass alloplast alone in human maxillary sinuses. The results demonstrated the highest amounts of newly deposited bone and maximum cellular activity in osteoblasts in the combination grafting group. Yadav et al. made a comparison between the clinical outcomes of guided tissue regeneration with collagen membrane only or collagen membrane covering autogenous bone substitute (test group I) and autogenous bone combined with bioactive bone glass (test group II) in intraosseous defects. Six months later, significant improvements in clinical parameters accompanied by defect healing and regeneration were detected in all groups with significant higher improvement in both test groups [7].

The implant survival rate and success rate of the two groups in the present study were 100% at 6 months' follow-up period. Survival rates of the implants inserted in our study were similar to that of Garcez‐Filho and colleagues [24], Blus and coauthors [25], and Annibali et al. [26], where implants were inserted at the same visit of ridge split with reported survival rate ranging between 91.7% and 100% and success rate ranging from 88.2% to 100% with follow-up duration from 1 to 10 years. Success/survival rate in the current study could not be determined because the maximum observation interval was only 6 months.

In present research, the mean buccopalatal width gain radiographically from baseline to 6 months after ridge splitting was found to be 3.61 ± 0.42 mm and 6.42 ± 1.38 mm for control and study groups, respectively. The results of the control group were consistent with Blus and colleagues [25], Simion et al. [4], and Scipioni and coauthors [5], whereas the results of the study group were higher than results of previously mentioned studies. Blus et al. performed ridge splitting procedures accompanied by simultaneous implants in maxilla and mandible. A total of 230 implants were inserted, with a bone width gain of around 2.5 to 4.0 mm [25]. Simion et al. [4] and Scipioni et al. [5] reported 1 to 4 mm gain in ridge width after the split‐crest technique and simultaneous immediate implants with successful osseointegration. In addition, short-term noncomparative studies of Santagata et al. [27] and Albanese et al. [28] revealed 3.25 to 3.5 mm gain in the ridge width following maxillary lateral expansion utilizing ridge split approach. Previously published systematic reviews have recorded 3.2 to 4.1 mm increase of the ridge after splitting of maxilla [29, 30].

The buccal and palatal plates are composed of the native bone, while the mesial and distal plates are composed of the bone graft substitute; thus variable remodeling and resorption pattern is observed [31]. In our study, the measurements of crestal bone resorption consisted of two values of bone loss: mesiodistal resorption and labiopalatal resorption.

In two publications of Tang et al. [32] and Bassetti et al. [33] assessing mesiodistal bone resorption around implants placed simultaneously with ridge splitting, greater bone resorption was detected in the first 6 months postsurgically with mean mesiodistal bone loss of 1.61 ± 0.91 mm (Tang et al.) and 1.19 ± 1.01 mm (Bassetti et al. [33]). The results of the present study were close to the previously mentioned studies, with mean mesiodistal bone loss of 1.37 ± 0.09 mm and 1.02 ± 0.02 mm for control and study groups, respectively, after 6months postoperatively. The current outcomes revealed that the mesiodistal bone loss in the control group was significantly higher than the study group; this could be contributed to the progressive resorption of autogenous bone graft. These results are in consistency with the studies of Cosso et al. [34] and Pereira et al. [35] who reported that the combination of autogenous bone graft and synthetic biomaterials is an effective substitute to stimulate new bone deposition and osseointegration and to reduce bone resorption.

The outcomes regarding labial bone loss in our study were found to be 1.55 ± 0.10 mm in the control group and 1.51 ± 0.07 mm in the study group. All these results were close to those reported by Jensen et al. [36] and Mounir et al. [31] who measured the mean amount of labial bone loss in the ridge splitting cases with simultaneous insertion of implants at 6 months postoperatively. They recorded that the amount of marginal bone resorption ranged from 1.5 to 3.5 mm. This high rate of labial bone resorption was attributed to stripping of the periosteal attachment, reduced blood supply, the diminished nourishment, the trauma caused by the cutting and splitting procedures itself, and the remodeling process of the thin labial cortical bone [31, 36].

Limitations of this study include small sample size, short follow-up period, the need for second reentry surgery, and postoperative histological assessment to confirm the quality and quantity of newly formed bone and the healing nature.

However, to the best of our knowledge, this is the first study to compare clinical outcomes of ridge splitting and simultaneous implant placement with autogenous bone or autogenous bone combined with bioactive glass in horizontal ridge defects. Further studies with a larger sample size and long-term observations would correspond with the findings presented here.

## 5. Conclusions

Within the limitations of the current study, 1 : 1, in volume, mixture of bioactive glass and autogenous bone graft has better outcomes compared to autogenous bone alone in the reconstruction of maxillary anterior horizontal ridge defects using alveolar ridge splitting accompanied by simultaneous implant placement.

## Figures and Tables

**Table 1:** Demographic data.

Variable	Control group	Study group
Age (years)		
Mean ± SD	38.64 ± 8.61	36.18 ± 6.89
Median (range)	39	38

Gender (*n* (%))		
Male	6 (54.54)	8 (72.72)
Female	5 (45.45)	3 (27.27)

**Table 2:** Mean bone width at baseline and 6 months postoperatively.

Test period	Mean bone width		Independent samples test	*p*-value
	Control group X ± SD	Study group X ± SD		
Preoperative	4.05 ± 0.53	3.72 ± 0.60	—	—
6 months	7.67 ± 0.55	10.14 ± 1.36	Sig. (2-tailed) = 0.00^*∗*^	*p* < 0.001^*∗*^
Paired *t*-test	*t* = −33.10	*t* = −17.90	—	—
	Sig. (2-tailed) = 0.00^*∗*^*p* < 0.001	Sig. (2-tailed) = 0.00^*∗*^*p* < 0.001	—	—

X ± SD: mean ± standard deviation; ^*∗*^statistical significant difference.

**Table 3:** Mean mesiodistal bone height at baseline and 6 months postoperatively.

Test period	Mean mesiodistal bone height		Independent samples test	*p*-value
	Control group X ± SD	Study group X ± SD		
Baseline	17.12 ± 2.30	17.70 ± 2.98	—	—
6 months	15.74 ± 2.42	16.67 ± 2.97	Sig. (2-tailed) = 0.00^*∗*^	*p* < 0.001^*∗*^
Paired *t*-test	*t* = 15.04	*t* = 41.37	—	—
	Sig. (2-tailed) = 0.00^*∗*^*p* < 0.001	Sig. (2-tailed) = 0.00^*∗*^*p* < 0.001	—	—

X ± SD: mean ± standard deviation; ^*∗*^statistical significant difference.

**Table 4:** Mean labial bone height at baseline and 6 months postoperatively.

Test period	Mean labial bone height		Independent samples test	*p*-value
	Control group X ± SD	Study group X ± SD		
Baseline	17.12 ± 2.30	17.70 ± 2.98	—	—
6 months	15.56 ± 2.47	16.18 ± 3.09	Sig. (2-tailed) = 0.76	*p* > 0.001
Paired *t*-test	*t* = 14.18	*t* = 20.21	—	—
	Sig. (2-tailed) = 0.00^*∗*^*p* < 0.001	Sig. (2-tailed) = 0.00^*∗*^*p* < 0.001	—	—

X ± SD: mean ± standard deviation; ^*∗*^statistical significant difference.

## Data Availability

The data are available upon request.
